# Stem Nematode Counteracts Plant Resistance of Aphids in Alfalfa, *Medicago Sativa*

**DOI:** 10.1007/s10886-014-0504-3

**Published:** 2014-09-28

**Authors:** Ricardo A. Ramirez, Lori R. Spears

**Affiliations:** Department of Biology, Utah State University, 5305 Old Main Hill, Logan, UT 84322 USA

**Keywords:** *Acyrthosiphon pisum*, *Ditylenchus dipsaci*, Host plant resistance, Plant pathogen, Aphididae, Peroxidase, Saponin

## Abstract

Plants are exploited by a diverse community of insect herbivores and phytopathogens that interact indirectly through plant-mediated interactions. Generally, plants are thought to respond to insects and pathogens through different defensive signaling pathways. As plants are selected for resistance to one phytophagous organism type (insect *vs.* pathogen) in managed systems, it is not clear how this selection may affect community interactions. This study examined the effect of nematode-resistant varieties on aphid (*Acyrthosiphon pisum*) suppression, and then determined how infection by the stem nematode, *Ditylenchus dipsaci*, mediated ecological effects on aphids and on plant defense proteins. Four alfalfa (*Medicago sativa*) varieties were selected with resistance to nematodes only (+,−), aphids only (−,+), nematodes and aphids (+,+), and susceptibility to nematodes and aphids (−,−). Field and greenhouse experiments were conducted to isolate the effect of nematode infection and aphid abundance on each variety. We found that varieties resistant to nematode, regardless of aphid resistance, had the lowest aphid counts, suggesting possible cross-resistance. Aphid abundance, however, increased when plants were exposed to nematodes. Resistant varieties were associated with elevated saponins but these compounds were not affected by insect or pathogen feeding. Concentrations of peroxidases and trypsin inhibitors, however, were increased in nematode resistant varieties when exposed to nematodes and aphids, respectively. The patterns of plant defense were variable, and a combination of resistance traits and changes in nutrient availability may drive positive interactions between nematodes and aphids aboveground.

## Introduction

The recognition that plants host a diverse community of phytophagous organisms and mediate community interactions has led to a surge in studies investigating the interactions between herbivorous insects and plant parasitic nematodes (McCarville et al. 2012, 2014; Wondafrash et al. 2013). In particular, studies have begun to evaluate the plant-mediated interactions between insects and nematodes. Insect feeding and nematode infection can induce various plant responses, namely plant defense compounds, and the resulting changes in a plant resource can alter nutrient availability, or the behavior and/or the fitness of the subsequent phytophagous organism (Bezemer et al. 2004; Kaplan et al. 2009; Manninen et al. 1998; van Dam 2009). A review of plant-mediated nematode and insect interactions showed that nematode-insect interactions may be positive, neutral, or negative but that feeding habits (*e.g.*, piercing-sucking *vs.* chewing mouthparts; migratory *vs.* sedentary endoparasitic) in part mediate these interactions (Wondafrash et al. 2013). In general, nematodes appear to neutrally and negatively affect piercing-sucking arthropods (*e.g.*, reducing aphid fecundity and plant attractiveness to aphids), while chewing arthropods (*e.g.*, caterpillars) have neutral to positive interactions with nematode infected plants (Alston et al. 1991; Carter-Wientjes et al. 2004; Kaplan et al. 2008, 2009; Olson et al. 2008). The majority of plant parasitic nematodes feed belowground on roots, and understandably much of what we know comes from interactions between these root feeders and aboveground insects. Studies on the interactions between aboveground feeding stem nematodes and insects are lacking and would contribute to the growing literature and knowledge of mechanisms driving nematode-insect interactions.

Alfalfa stem nematodes, *Ditylenchus dipsaci,* are agriculturally important migratory endoparasites that harm *Medicago sativa* (alfalfa) and other plant hosts (Duncan and Moens 2006). Unlike many soil-dwelling plant parasitic nematodes, stem nematodes have free living stages in the soil that infect the crown buds of alfalfa but are carried in growing plant tissue aboveground and cause swollen nodes, and shortened internodes (Bohart et al. 1976). Furthermore, stem nematodes can migrate to apical regions of shoots and enter plant tissue through stomata, or penetrate directly into stems and leaf axils (Duncan and Moens 2006). Similar to root-knot nematodes, stem nematodes have the ability to manipulate or suppress host plant responses when developing in the plant (Duncan and Moens 2006; Gheysen and Mitchum 2011). Stem nematodes are pronounced in early spring when temperatures are cool (<20 °C) (Duncan and Moens 2006) and interact with young plants before insect herbivores become active. The pea aphid, *Acyrthosiphon pisum*, occurs from late-spring to mid-summer on shared host plants and is abundant, therefore, making it a good model herbivore to investigate nematode-insect interactions.

Induced plant responses toward phytophagous organisms rely on the plant hormones salicylic acid (SA), jasmonic acid (JA), and ethylene (ET) (Pieterse et al. 2012). In general, SA mediates induction of systemic acquired resistance (SAR) in plants toward plant pathogens, while the JA and ET pathways are induced by insect feeding (Pieterse and van Loon 1999). Current literature, however, finds that these responses can be tailored toward the type of herbivory. For example, necrotrophic pathogens and chewing insects tend to signal the JA pathway, while biotrophic pathogens and phloem-feeding insects tend to signal the SA pathway (Bari and Jones 2009; Smith and Boyko 2006). Moreover, SA can suppress JA signaling (Spoel et al. 2003) but there is a considerable amount of cross-talk among these signaling pathways (Bostock 2005; Pieterse et al. 2012). The complexity of this signaling leads to an array of defense responses against pathogens and insects, including the production of defensive proteins, toxins, and volatiles (Bezemer and van Dam 2005; Dicke et al. 2009; Kaplan et al. 2008). In particular, several proteins (*e.g.*, chitinase, peroxidase, polyphenol oxidase, and trypsin inhibitor) have been implicated in the toxicity, disruption of digestion, and decrease in nutritional value of plants toward nematodes and insects (Bi et al. 1997a, b; Felton et al. 1992; Kabir et al. 2006; Kramer and Muthukrishnan 1997; Ryan 1990; Zhu-Salzman et al. 2008). Defense proteins can be linked with the SA (chitinase and peroxidase) and JA (polyphenol oxidase and trypsin inhibitors) pathways more specifically (Barto and Cipollini 2005; and references therein). These responses may lead to trade-offs in defense when plants are attacked by pathogens *vs.* insects, and may help explain the outcomes of nematode-insect interactions. Interestingly, however, an overlap of plant responses to pathogens and aphids has been observed. For example, aphid feeding can lead to the up-regulation of pathogen-response (PR) RNA genes and production of defense proteins that lead to antixenosis (Girousse and Bournoville 1994; Smith and Boyko 2006; Walling 2000). In addition, plants may respond to aphids with localized cell death at feeding sites, similar to the hypersensitive response of plants toward plant pathogens (Boyko et al. 2006; Lyth 1985).

Considering the general mechanisms of plant resistance for pathogens and insects (*e.g.*, SA *vs.* JA signaling), tradeoffs may exist when selecting plants that are resistant to one phytophagous organism *vs.* another. Nematode-resistant alfalfa is one tactic used to suppress stem nematode populations. Alfalfa is a perennial, tetraploid legume with polysomic inheritance and broad genetic variation among commercial varieties (Maureira and Osborn 2005). Breeding alfalfa and understanding the mechanisms directed at resistance is complex given that varieties are synthetic populations created by crossing a number of parents of selected genotypes (Maureira and Osborn 2005). Saponins, a class of glycosides, are involved in herbivore suppression, and some alfalfa has been selected with elevated saponin content, but the effects on insects and pathogens are idiosyncratic and not consistent as resistance is in other systems. In tomato, for example, the *Mi-1 R* protein requiring SA signaling leads to resistance toward root-knot nematodes and cross-resistance to aphids and whiteflies (Nombela et al. 2003; Rossi et al. 1998; Vos et al. 1998). Plants without the *Mi-1* gene are more susceptible to aphids but only when JA signaling is blocked (Bhattarai et al. 2007). As plant breeding and selection for resistance is geared toward particular phytophagous organism types, the possibility exists that host plants are silenced in particular defenses or that cross resistance is established.

What complicates these interactions is that nematodes and insects have ways to evade various plant resistance traits and also induce plant responses. Nematodes, for example, have enzymes that aid in cell wall degradation, protect against reactive oxygen species (Jones et al. 2004), and effectors that suppress SA and JA production (Haegeman et al. 2012). Aphids avoid plant defenses with their saliva by plugging disrupted cells and creating a feeding tube and using watery saliva to prevent feeding site occlusion (Walling 2008). In addition, some aphids reduce JA-regulated RNAs by increasing SA pathways (Prado and Tjallingii 2007; Thompson and Goggin 2006). Therefore, on a shared host plant, it is possible for one organism to “prime” plant responses in a way that affects the second herbivore. Nematode infected plants, for example, have been shown to reduce caterpillar performance because they elevate levels of phenolics and glucosinolates in *Brassica* (van Dam et al. 2005) and increase caterpillar performance in tobacco by interfering with nicotine synthesis (Kaplan et al. 2008).

Field and greenhouse trials were conducted to examine how alfalfa varieties with varied resistance and stem nematode infection impact aphid abundance and plant defense responses. In particular, varieties were selected with resistance to nematodes only (PGI-437), aphids only (Rugged), both nematodes and aphids (Sutter), and a susceptible variety (Vernal). The objectives of this study were to determine (1) how selection of resistant varieties impact aphid populations, (2) how aboveground nematodes affect aphid abundance, and (3) to gain insight into the plant defense responses toward stem nematode and aphid among resistant varieties.

## Methods and Materials

### Evaluating Nematode-Aphid Interactions

A greenhouse trial was conducted at Utah State University’s Research Greenhouse Complex in Logan, UT, USA. In each of two years (2011–2012), we evaluated the presence of nematodes on aphid abundance and plant defense protein concentration for each of four alfalfa varieties varying in resistance to nematodes and aphids. Using the National Alfalfa & Forage Alliance’s pest resistance rating guides (www.alfalfa.org), we chose varieties that crossed stem nematode resistance (+, −) with pea aphid resistance (+, −). Given the high level of genetic variation of *M. sativa*, resistance corresponds to % resistant plants, where highly resistant (HR), resistant (R), moderately resistant (MR), and low resistant or susceptible (S) ratings are represented by >50 %, 31–50 %, 15–30 %, and <14 % resistant plants, respectively (Table 1). Here, we selected Vernal (susceptible to nematodes and aphids), PGI-437 (resistant to nematodes but not aphids), Rugged (resistant to aphids but not nematodes), and Sutter (resistant to nematodes and aphids) varieties.

**Table 1:** Resistance ratings for alfalfa varieties (NAFA 2014), where highly resistant (HR), resistant (R), moderately resistant (MR), and susceptible (S) ratings are represented by >50 %, 31–50 %, 15–30 %, and <14 % resistant plants, respectively. For this study, we lumped highly resistant and resistant varieties into a resistant category (+) and moderately resistant and susceptible varieties into a susceptible category (−)

Variety	Nematode Resistance	Aphid Resistance
Pershing^b^	R(+)	R(+)
Rugged^ab^	MR(−)	HR(+)
Vernal^ab^	S(−)	S(−)
PGI-437^a^	R(+)	MR(−)
Sutter^a^	R(+)	R(+)

Varieties examined in the greenhouse are indicated by ^a^; field varieties are indicated by ^b^

Each of the four varieties was subjected to nematode treatments (+,−) representing eight unique treatments each replicated 4 times for each of two trials (*N* = 64). Our experimental unit was a microcosm, consisting of an 800-ml-capacity pot (8.9 × 8.9 × 8.9 cm) filled with soil (Sunshine Mix #2 potting soil). Each microcosm was seeded with 12 alfalfa seeds (at standard field seeding rates), watered once a week, fertilized with 1 g of Osmocote (10N-10P-10K), and maintained at 24/21 °C day/night temperatures. After 2 wk of plant growth, each nematode treatment pot was inoculated with 30 field collected stem nematodes (*Ditylenchus dipsaci*). Nematodes were extracted by chopping infested plant stems in water, and then collected with a pipette before transferring them to plants (Hanna and Hawn 1965). Symptomatic plants had shortened and swollen internodes compared to non-symptomatic plants. One week following nematode inoculation, each microcosm received 5 laboratory-reared (23 °C; 12 h L/D cycle) aphids. Aphids were confined using an inverted cup (Solo, 473-ml clear plastic cup, Highland Park, IL, USA) with mesh replacing the bottom and 40 cm^2^ sides (vents on opposite sides) of the cup. The cup rim was fixed to the soil surface. After 2 wk, aphids were counted, and alfalfa stems were clipped at the base from 13 cm tall vegetative stage plants and stored in the freezer to measure plant defense proteins at a later date (see *Plant defense bioassays* section below).

### Evaluating Resistant Varieties on Aphid Abundance in the Field

A subsequent field experiment was conducted at Utah State University’s Greenville Research Station in Logan, UT, USA. We examined alfalfa plots in each of 3 yr (2011–2013) for the abundance of aphids and plant defense protein concentration on each of three alfalfa varieties. We selected Vernal (susceptible to nematodes and aphids), Rugged (resistant to aphids but not nematodes), and Pershing (resistant to nematodes and aphids). Each of these varieties was planted in 5 replicate plots (1.5 × 3 m; 15 plots total) in a completely randomized design. Plots within rows were separated by 1.2 m of fallow buffer, while adjacent plots were separated by 0.9 m of fallow buffer. A single variety was seeded in a plot at 9 kg seed per 4046.9 m^2^. The option for including a nematode-only resistant variety in the field was limited because this variety had low winter dormancy, was a poor performer in our area, and therefore not included in our field experiment. It is important to note that stem nematodes have not been detected at this field site and are not prevalent in Cache County, UT, USA.

When alfalfa was ~30.5 cm tall, mesh sleeves (tulle fabric) were placed over four stems per plot and closed using a twist-tie. Two sleeves from each plot were randomly selected to house 20 aphids each. The remaining two sleeves were left untreated (no aphids) as controls. Selected plants were not used in subsequent years. After 2 wk, plant stems from early-bud stage plants were clipped and immediately placed on ice to process aphid counts and for later analysis of plant defense protein concentration.

### Plant Defense Bioassays

Leaves collected from stems of the field and greenhouse experiments were analyzed for levels of chitinase (CHI), peroxidase (POD), polyphenol oxidase (PPO), and trypsin inhibitor (TI) following modified methods from Cipollini et al. (2004). POD, CHI, and PPO activity were measured using a microplate reader (Biotek EPOCH, Winooski, VT, USA). Briefly, POD and PPO activity was determined following the oxidation of guaiacol and caffeic acid, respectively, for 1 min at 470 nm. CHI activity in soluble protein extracts was examined by assessing the hydrolysis of *p*-nitrophenyl-β-N-acetylglucosaminide measured at 405 nm. TI activity was measured by examining the diffusion of protein extracts through a trypsin-containing agar followed by staining with N-acetylphenylalanine napthyl ester and *o*-dianisidine. TI concentration was determined using a standard curve made with soybean trypsin inhibitor, expressed as μg trypsin inhibitor/g protein.

Saponin activity was determined by using modified procedures of Kendall (1964). Briefly, foaming capacities of alfalfa plant tissues were measured by grinding 0.50 g (greenhouse samples) and 1.25 g (field samples) of alfalfa into 65 ml and 130 ml of distilled water, respectively, with a food blender for 2 min. The extract was poured into a graduated cylinder (100 ml for greenhouse samples and 250 ml for field samples). After the extract settled for 2 min, the cylinder was shaken vigorously for 10 sec to eliminate trapped air bubbles before the final amount of crude saponin level (foam) was recorded.

### Statistical Analyses

The greenhouse microcosm experiments (TRIAL 1 and TRIAL 2) were analyzed together within a 2 × 2 × 2 factorial design having aphid resistance (+,−), nematode resistance (+,−), and nematode application (+,−). Aphid counts from the field experiment were analyzed with two factors, TIME (for each of the 3 yr) crossed with alfalfa VARIETY (Vernal, Rugged, and Pershing). For greenhouse and field experiments, aphid counts were log transformed, and generalized linear model (GLM) was used for analysis. GLM was followed by Tukey’s *post hoc* test of VARIETY given no significant interaction (TIME×VARIETY) and a significant VARIETY main effect. We also examined whether there was a correlation between aphid abundance and each plant defense protein concentration and nematode symptomatic plants. No relationship between aphid abundance and these factors was evident (*r* ^*2*^ < 0.11) and were not examined further.

Plant defense protein data from the greenhouse experiment were analyzed using a three-way ANOVA with alfalfa VARIETY, APHID treatment (+,−), and NEMATODE treatment (+,−) as factors. Plant defense protein data from the field experiment were first analyzed using a three-way ANOVA with TIME, alfalfa VARIETY, and APHID treatment as factors. To better interpret TIME interactions for PPO, each year was analyzed separately using a two-way ANOVA that included VARIETY and APHID treatment as factors. Significant differences in main effects were followed by Tukey’s *post hoc* tests and differences in interaction terms were examined with step-down Bonferroni adjustments.

Analyses of aphid counts from the field study and greenhouse experiment were analyzed using SYSTAT (version 13.0; SPSS, Chicago, IL, USA) software; plant defense protein data were analyzed using SAS (version 9.3; SAS Institute Inc., Cary, NC, USA).

## Results

### Effects of Resistant Varieties and Nematodes on Aphid Abundance in the Greenhouse

**Fig. 1:**
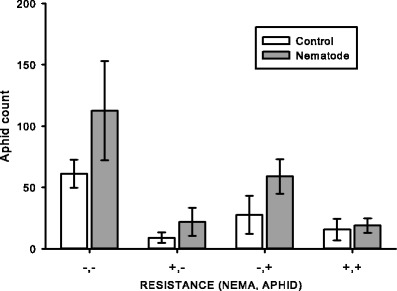
From the greenhouse experiment (2011–2012), aphid counts on susceptible (−,−; Vernal), nematode resistant (+,−; PGI-437), aphid resistant (−,+; Rugged), and combined aphid and nematode resistant (+,+; Pershing) varieties. *White bars* represent control treatments without nematodes and *grey bars* represent treatments with nematodes. Values are means ±1SE

At the start of the study, nematode treated pots showed symptoms of infection on 44 % of plants ±9 SE in the susceptible variety (Vernal), 33 % ±6 in the nematode resistant variety (PGI-437), 48 % ±8 SE in the aphid resistant variety (Rugged), and 31 % ±6 in the combined resistant variety (Sutter). In general, aphid abundance was lower when varieties had nematode resistance regardless of plants being resistant to aphids (*F*_1,55_ = 25.669, *P* < 0.001) (Fig. 1). When plants were exposed to stem nematodes we found an increase in aphid abundance (*F*_1,55_ = 8.234, *P* = 0.006). The effect of nematodes by variety was variable; the percent increase of aphid abundance for the susceptible (Vernal), nematode resistant (PGI-437), aphid resistant (Rugged), and combined resistance (Sutter) varieties were 84 %, 69 %, 88 %, and 20 %, respectively.

### Response of Resistant Varieties to Aphids and Nematodes in the Greenhouse

**Fig. 2:**
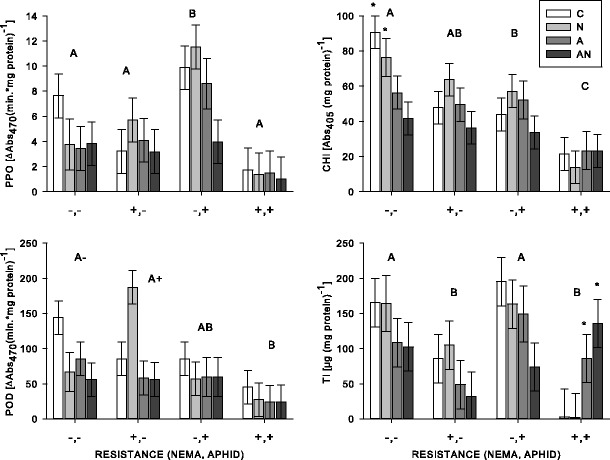
From the greenhouse experiment (2011–2012), plant defense protein activity in susceptible (−,−; Vernal), nematode resistant (+,−; PGI-437), aphid resistant (−,+; Rugged), and combined aphid and nematode resistant (+,+; Sutter) varieties. *Bar color* indicates treatment type (C = control, N = nematode application, A = aphids present, AN = aphids and nematode application). Values are means ±1SE. *Capital letters* that are different from each other represent significant (*P* < 0.05) main effects of variety (one letter per variety); an *asterisk* indicates significant differences among treatments within a variety; minus (−) and plus (+) *symbols* indicate varieties that are different from the variety resistant to nematodes and aphids (Sutter) when nematodes were not applied and when nematodes were applied, respectively. Main effects of aphids (for PPO, CHI, and POD) are not shown

Bioassays for each of the four defense proteins revealed variation among the varieties. The susceptible variety (Vernal) had the highest level of CHI (VARIETY: *F*_3,45_ = 15.03, *P* < 0.001) and the variety (Rugged) resistant to aphids had the highest level of PPO (VARIETY: *F*_3,46_ = 10.75, *P* < 0.001; all Tukey comparisons *P* < 0.02). Both of these varieties also had high levels of TI (VARIETY: *F*_3,45_ = 6.56, *P* = 0.001). Alternatively, the variety (Sutter) with combined resistance to nematodes and aphids had lower concentrations of PPO than the variety (Rugged) resistant to aphids (Tukey *P* < 0.001) and CHI (Fig. 2).

When plants were exposed to aphids, PPO concentrations decreased compared to plants without aphids (APHID: *F*_1,46_ = 4.56, *P* = 0.038). Similarly, we found a drastic decrease in CHI for the susceptible variety (Vernal) when exposed to aphids (Bonferroni *P* = 0.013), but the magnitude of decrease by aphids for other varieties was small to non-existent (VARIETY: *F*_3,45_ = 15.03, *P* < 0.001; APHID: *F*_1,45_ = 6.52, *P* = 0.014; VARIETY×APHID: *F*_3,45_ = 2.93, *P* = 0.044) (Fig. 2). The presence of aphids did, however, elevate TI in the combined resistant variety (Sutter) (Bonferroni *P* = 0.056), while aphids decreased TI concentrations in all other varieties, and apparently drove this interaction (VARIETY×APHID: *F*_3,45_ = 5.63, *P* = 0.002) (Fig. 2).

Nematodes did not alter PPO, TI, or CHI levels among the varieties (all main and interaction effects involving NEMATODE: *P* > 0.05). Similarly POD concentration was not altered by nematodes for the aphid resistant variety (Rugged) or combined resistant variety (Sutter). However, an interaction apparently driven by the presence of nematodes reduced POD in the susceptible variety (Vernal), and elevated POD concentrations in the nematode resistant variety (PGI-437) (VARIETY: *F*_3,45_ = 6.14, *P* = 0.001; APHID: *F*_1,45_ = 7.82, *P* = 0.008; VARIETY×NEMATODE: *F*_3,45_ = 3.07, *P* = 0.037) (Fig. 2).

**Fig. 3:**
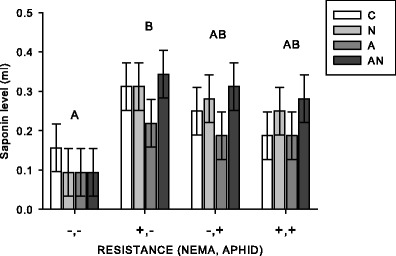
From the greenhouse experiment (2011–2012), crude saponin activity in susceptible (−,−; Vernal), nematode resistant (+,−; PGI-437), aphid resistant (−,+; Rugged), and combined aphid and nematode resistant (+,+; Sutter) varieties. *Bar color* indicates treatment type (C = control, N = nematode application, A = aphids present, AN = aphids and nematode application). Values are means ±1SE. *Different capital letters* represent a significant (*P* < 0.05) main effect of variety

The susceptible variety had the lowest saponin levels (VARIETY: *F*_3,4_ = 7.13, *P* = 0.044) but had similar levels to the combined resistant variety (*P* = 0.159), was not significantly different from the aphid resistant variety (*P* = 0.082), and was significantly different from the nematode resistant variety (*P* = 0.039) (Fig. 3). Saponins were not affected by nematodes (NEMATODE: *F*_1,12_ = 2.40, *P* = 0.147) or aphids (APHID: *F*_1,12_ = 0.27, *P* = 0.615).

### Effects of Resistant Varieties on Aphid Abundance in the Field

**Fig. 4:**
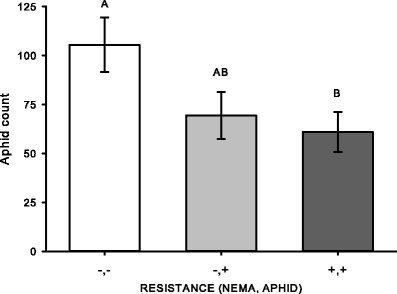
From the field experiment (2011–2013), aphid counts on susceptible (*white bar*; Vernal), aphid resistant (*light grey bar*; Rugged), and combined aphid and nematode resistant (*dark grey bar*; Pershing) varieties. Values are means ±1SE. *Different capital letters* indicate significant differences (*P* < 0.05)

Changes in aphid abundance were dependent on the host variety, and these differences were constant over time (TIME: *F*_1,39_ = 2.038, *P* = 0.144; VARIETY: *F*_2,39_ = 9.284, *P* = 0.004; TIME×VARIETY: *F*_2,39_ = 1.933, *P* = 0.158). A closer look revealed that the variety resistant to nematodes and aphids (Pershing) had the lowest abundance of aphids in the field (*P* = 0.049) (Fig. 4). This parallels aphid abundance on nematode resistant varieties in the greenhouse. The aphid abundance was 25 % lower on the aphid resistant variety (Rugged) than on the susceptible variety, however, this was not statistically significant (*P* = 0.267).

### Response of Resistant Varieties to Aphids in the Field

**Fig. 5:**
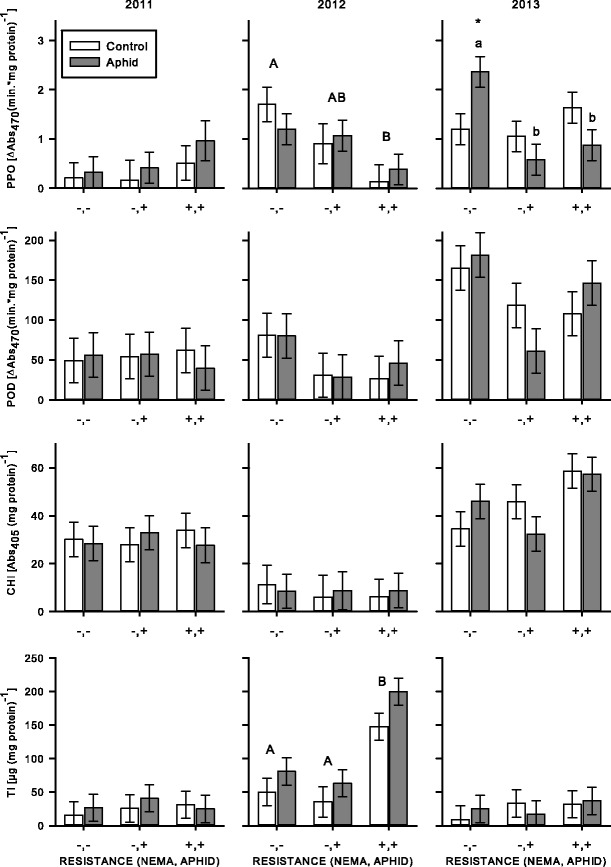
From the field experiment, plant defense activity in susceptible (−,−; Vernal), aphid resistant (−,+; Rugged), and combined aphid and nematode resistant (+,+; Pershing) varieties. *White bars* represent control treatments without aphids and *grey bars* represent treatments with aphids. Values are means ±1SE. *Different capital letters* represent a significant (*P* < 0.05) main effect of variety; *different lowercase letters* represent differences among treatments with the same shade bar; an *asterisk* indicates a difference from the control within a variety. Main effects of variety (for POD) and year (for POD, CHI, and TI) are not shown

A bioassay of PPO revealed that aphids induced elevated levels of PPO, but induction varied among varieties and years (TIME×VARIETY×APHIDS: *F*_4,39_ = 2.91, *P* = 0.034). This was contrary to the decreased PPO levels in the greenhouse experiment. To describe this interaction we analyzed each year separately. Newly established alfalfa varieties (2011) with aphids showed consistent increases in PPO (APHIDS: *F*_1,8_ = 0.06, *P* = 0.065) (Fig. 5). The response toward aphids, however, was not seen in the following year. In mature plants (2013), an interaction between the presence of aphids and among varieties was apparently driven by the susceptible variety (Vernal) having an elevated PPO concentration when aphids were present compared to the aphid resistant and combined resistant varieties where aphid feeding decreased PPO (APHIDS×VARIETY: *F*_2,12_ = 10.44, *P* = 0.002).

Aphids did not appear to affect POD, CHI, or TI concentrations in the field (all main and interaction effects involving APHIDS: *P* > 0.05). More so, differences in POD varied among varieties and year. The susceptible variety (Vernal) had higher concentrations of POD than the aphid resistant variety (Rugged) (VARIETY: *F*_2,12_ = 3.91, *P* = 0.049; Tukey *P* = 0.045). As varieties matured (3 year establishment), POD levels were elevated compared to newly established alfalfa plants (TIME: *F*_2,48_ = 16.20, *P* < 0.001; Tukey comparisons *P* < 0.001) (Fig. 5). Induced responses to aphid were not evident, but in 2013 CHI levels were at their highest compared to other years (TIME: *F*_2,44_ = 37.47, *P* < 0.001; all Tukey comparisons *P* < 0.001). In addition, in 2012, TI was elevated in the combined resistant variety (Pershing) compared to the other varieties (TIME: *F*_2,47_ = 26.45, *P* < 0.001; VARIETY: *F*_2,12_ = 5.38, *P* = 0.021; TIME×VARIETY: *F*_4,47_ = 7.63, *P* < 0.001; all Bonferroni comparisons *P* < 0.001) (Fig. 5).

**Fig. 6:**
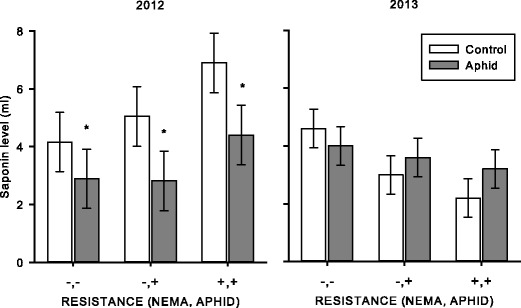
From the field experiment, crude saponin activity in susceptible (−,−; Vernal), aphid resistant (−,+; Rugged), and combined aphid and nematode resistant (+,+; Pershing) varieties. *White bars* represent control treatments without aphids and *grey bars* represent treatments with aphids. Values are means ±1SE. An *asterisk* indicates a difference from the control within a variety

In 2012, the constitutive saponin defenses also were elevated in the resistant varieties compared to the susceptible variety, an effect we found in the greenhouse experiment. However, this was not witnessed in mature plants in 2013 (TIME: *F*_1,6_ = 4.04, *P* = 0.091; VARIETY: *F*_2,12_ = 0.33, *P* = 0.728; TIME×VARIETY: *F*_2,6_ = 5.39, *P* = 0.046). Upon further investigation of this interaction, in 2012 the saponin content was reduced in the presence of aphids, and this was not seen in mature plants in 2013 (APHIDS: *F*_1,12_ = 2.78, *P* = 0.121; TIME×APHIDS: *F*_1,6_ = 6.60, *P* = 0.042; 2012 Bonferroni comparisons *P* = 0.028) (Fig. 6).

## Discussion

Nematodes can alter the performance of arthropods and other plant pathogens on shared host plants (Hawn and Hanna 1967; Wondafrash et al. 2013). Here, we found that stem nematodes increased aphid abundance on alfalfa. This is in contrast to the studies that tested the effects of root-feeding nematodes on aphid abundance aboveground (examples in McCarville et al. 2012; Wondafrash et al. 2013). One of a few studies examining stem nematodes investigated the impact of stem nematodes on bacterial wilt (*Corynebacterium insidiosum*) and found that nematodes also can increase the susceptibility of alfalfa varieties to this bacterium (Hawn and Hanna 1967). An example on melon plants shows that other plant pathogens also can enhance aphid abundance (Moran 1998). What we know from root feeding nematodes is that their negative effect on aphids aboveground may be due to a decrease in the availability of amino acids in phloem sap (Bezemer et al. 2005). In our study, we found that overall protein concentration increased in plants with stem nematodes (mean 0.66 mg N/ml ± 0.09 SE) compared to the absence of nematodes (mean 0.46 mg N/ml ± 0.09 SE). We were careful not to confound protein from nematodes with plant protein by avoiding stem tissue where nematodes primarily reside and by selecting leaf tissue for assays. Thus, our protein concentrations suggest that nitrogen based nutrients (*e.g.*, amino acids) play a role in nematode-insect interactions and may have contributed to increasing aphid abundances in this study. Given the complexity of responses we found with the plant defense proteins, it is a worthy endeavor to evaluate how stem nematodes affect plant nutrition from root to shoot.

Plant parasitic nematodes have the ability to suppress SA and JA signaling pathways that suppress plant defenses (Haegeman et al. 2012). These changes may provide a more suitable host for other phytophagous organisms. Stem nematodes previously have been shown to induce plant defenses in alfalfa. Interestingly, isoflavonoids and other metabolites were not changed in shoots where stem nematodes reside but rather increased in concentration in the roots (Edwards et al. 1995). Although root-lesion nematodes do not appear to affect isoflavonoids in alfalfa roots, they have been shown to increase phenylpropanoid and chitinase, among other mRNAs important to plant defense in roots (Baldridge et al. 1998). We found an elevated POD concentration in leaves when a nematode resistant variety was exposed to nematodes. Furthermore, nematodes were able to decrease the POD concentration in the leaves of the susceptible variety. Stem nematodes, however, did not affect other defense proteins we tested. The root accumulation of plant defenses as seen in these other studies may account for similar protein concentrations in leaves between nematode infected and non-infected alfalfa observed in our results. Another factor to consider for the low detection of induction by nematodes is that the duration of plant defense changes by nematodes appears to be brief. Plants infected with root-lesion nematodes had elevated concentrations of plant defenses within 12 h of infections, however, they were short lived, and the concentrations were decreased back to levels seen in susceptible varieties within 48 h (Baldridge et al. 1998). Alfalfa plants in our study were tested after 4 weeks of being exposed to nematodes. Therefore, stem nematode effects on plant defense proteins may have occurred early on but were not captured because the effects diminish quickly.

Our selection of proteins, two of which are considered to be related to the SA (CHI and POD) and JA (PPO and TI) pathways (Barto and Cipollini 2005; and references therein), was to help tease apart competing SA and JA pathways and determine any tradeoffs related to defense toward pathogen and insect. Although we did not see a decrease in plant defenses across varieties that would suggest the plant is more susceptible to aphids, nematodes may favorably alter nutrients and subsequent aphid feeding affected plant defense proteins. Aphids divert nitrogen from the apical growth of alfalfa to feeding sites (Girousse et al. 2005). Only recently have we begun to understand the molecular bases of aphid resistance in plants. In *Medicago truncatula*, for example, jasmonic acid has been implicated in R-gene mediated aphid resistance (Gao et al. 2007). More specifically, the AKR gene imparts resistance toward the bluegreen aphid (*Acyrthosiphon kondoi*) by elevating JA defenses (Gao et al. 2007). However, there is much variation among aphid species, and distinctive *Medicago* phenotypes are observed (Klingler et al. 2005; Walling 2008). In our study, aphids increased TI concentrations in varieties resistant to both nematodes and aphids. Similarly, the tobacco aphid up-regulates TI in tobacco plants. Across all alfalfa varieties, we found reduced PPO concentration with aphids, an effect encountered in sorghum where aphids down-regulate PPO (Smith and Boyko 2006). Interestingly, we found this to be opposite when evaluating alfalfa plants in the field. In contrast to sorghum plants, CHI concentration decreased with aphid feeding. POD was not affected by aphids in our study, but aphid feeding down-regulates POD in sorghum and *Arabodopsis,* and is up-regulated in celery (Smith and Boyko 2006). Given that aphids mostly decreased plant defense proteins in our study, aphids may be using various strategies to overcome alfalfa defenses (see Walling 2008). In the variety resistant to nematodes and aphids and where we see aphid abundance to be lowest, we find increases in TI proteins when exposed to aphids, and this may in part aid in this variety being a less suitable host.

The molecular mechanisms of plant defense for aphids are not fully understood but what is clear is that aphids in general activate multiple signaling pathways and that signaling is mediated by multiple compounds, as described above in several plant species (Smith and Boyko 2006 and examples therein). General models of aphid-plant interactions according to host plant resistance have been described where aphid-resistant plants show gene-for-gene recognition and defense signaling early on, and resistant and susceptible plants recognize aphid-specific cell damage (Smith and Boyko 2006). SA signaling has been closely linked with aphids, but more specific loci have been identified in resistance of *M. truncatula*, a close relative of *M. sativa*, to the blue alfalfa aphid (*A. kondoi*). In particular, a chromosome region neighboring resistance gene analogs has been mapped that encode the coiled-coil (CC)-NBS-LRR resistance proteins (Klingler et al. 2005). More recently, jasmonate ZIM (JAZ) was found to interact with jasmonate insensitive 1/MYC2 and inhibit JA-responsive gene expression (Bari and Jones 2009). Study of these specific genes and resistance regions is warranted to unravel the complexity of aphid-plant interactions and the inherent specificity among aphid species and their plant host.

Although saponin levels were not affected by nematodes or aphids, resistant varieties had higher saponin levels compared to the susceptible variety. There is evidence that high saponin levels negatively affect pea aphids by altering aphid probing behavior (Golawska 2007; Golawska et al. 2006; Pedersen et al. 1976). High saponin levels however, were not important in the suppression of the spotted alfalfa aphid (*Therioaphis maculate* Bruckton) or root-knot (*Meloidigyne hapla*) or stem nematodes (*D. dipsaci*) (Pedersen et al. 1976). This may in part help explain the general resistance of pea aphid in our study toward both nematode and aphid resistant varieties. Cross-resistance by other mechanisms has been seen in the soybean system where resistance toward the root nematode, *Heterodera glycines,* reduces the performance of aphids, *Aphis glycines* (McCarville et al. 2012). Increases in arthropod herbivore density in alfalfa have been shown to increase saponin levels (Pearson et al. 2008), but we did not find this effect in our study. Saponins are a complex of many compounds and specific saponin compounds (*i.e.*, zanhic acid tridesmoside and 3-GlcA, 28-AraRhaXyl medicagenic acid glycoside) appear to be more important in resistance than others (Golawska et al. 2006). It is possible that these particular saponins may be altered and may help explain the increased aphid abundance in the presence of nematodes but specialized bioassays for these compounds would be warranted.

The mechanisms for pest resistance in alfalfa are complex given the level of genetic variation within and among varieties. In alfalfa roots, resistant varieties may be elevated in certain defenses compared to susceptible varieties, but other defense indicators such as chitinase mRNAs were similar between resistant and susceptible varieties (Baldridge et al. 1998). Indeed, we found elevated saponins in resistant varieties compared to the susceptible variety, but only particular varieties expressed elevated defensive proteins. The susceptible variety was elevated in CHI and TI, and the aphid resistant variety was elevated in PPO and TI. Overall, there appears to be a lack of consistency with any particular defense strategy, and perhaps unique combinations of defenses are expressed for each variety. For example, some species of aphids are susceptible to high saponin concentrations while other aphid species are not (Golawska, 2007; Golawska et al. 2006; Pedersen et al. 1976), yet the mechanism targets the mouthparts for which this is similar for aphids. Alfalfa resiliency to respond to continual disturbances including routine harvest (wounding) periods in a season, herbivory, and environmental factors, may provide for more plasticity and perhaps unique methods for coping with these stressors in any given time. In our study, we found induced responses toward aphids for newly established plants and mature plants, but in 2012 induced responses were not evident. What we found, however, was that TI and saponin levels were elevated in this particular year. Indeed, environmental factors (*e.g.*, temperature, rainfall, drought, *etc.*) can cause stresses in plants leading to induced defenses that may interact with responses to herbivores. We did not identify specific linkages with abiotic factors (*i.e.*, temperature and relative humidity) that would explain our results in the field but other environmental factors cannot be ruled out. We were limited to only a single variety or two within a resistant variety type (stem nematode resistant, pea aphid resistant, or resistance to both) because of the lack of available commercial varieties with these particular resistance qualities. As we approach having a completed alfalfa genome available, we may better isolate particular genes and gene interactions involved in plant defense against pathogens and insects.

It has long been known that alfalfa resistance is complex, and this also is evident in the classification of resistant varieties to particular phytophagous organisms. Resistance is generalized at the population level where a highly resistant variety indicates that > 50 % of the population is resistant to the target organism. Interestingly, in our study the aphid resistant variety maintained a high abundance of aphids in the field, yet the plant quality did not appear to be affected in the way the susceptible variety showed yellowing and other signs of damage (pers. obs.). Therefore, some varieties classified as resistant may in fact be tolerant and still maintain high quality. Resistance in alfalfa is unlike other plant systems (*e.g.*, *Zea mays*) where resistance is more uniform within a variety. Therefore, enhanced genomic resources will be critical tools for understanding resistance and the trade-offs to defend against multiple phytophagous organisms.
